# Phosphorylation Modifications Regulating Cardiac Protein Quality Control Mechanisms

**DOI:** 10.3389/fphys.2020.593585

**Published:** 2020-11-12

**Authors:** Sumita Mishra, Brittany L. Dunkerly-Eyring, Gizem Keceli, Mark J. Ranek

**Affiliations:** ^1^Division of Cardiology, Department of Medicine, The Johns Hopkins University School of Medicine, Baltimore, MD, United States; ^2^Department of Pharmacology and Molecular Sciences, Johns Hopkins University, Baltimore, MD, United States

**Keywords:** phosphorylation, chaperones, proteasome, autophagy, ubiquitin enzymes, cardiac disease

## Abstract

Many forms of cardiac disease, including heart failure, present with inadequate protein quality control (PQC). Pathological conditions often involve impaired removal of terminally misfolded proteins. This results in the formation of large protein aggregates, which further reduce cellular viability and cardiac function. Cardiomyocytes have an intricately collaborative PQC system to minimize cellular proteotoxicity. Increased expression of chaperones or enhanced clearance of misfolded proteins either by the proteasome or lysosome has been demonstrated to attenuate disease pathogenesis, whereas reduced PQC exacerbates pathogenesis. Recent studies have revealed that phosphorylation of key proteins has a potent regulatory role, both promoting and hindering the PQC machinery. This review highlights the recent advances in phosphorylations regulating PQC, the impact in cardiac pathology, and the therapeutic opportunities presented by harnessing these modifications.

## Introduction

Hearts are tasked with an immense challenge of maintaining protein homeostasis (proteostasis) in the face of disease conditions stemming from genetic mutations or environmental stressors (Willis and Patterson, 2013; Henning and Brundel, 2017). Pathological stressors can produce non-functional, misfolding-prone proteins. The failure to remove these proteins in a timely manner can compromise the integrity of intracellular proteins and impair the contractile apparatus and organelles; this results in decreased cell survival and function, culminating in the development of heart failure (Wang and Robbins, 2006; Wang et al., 2008). Indeed, many forms of heart disease present with the accumulation of ubiquitinated proteins and/or protein aggregates: hallmarks of inadequate or impaired PQC (Weekes et al., 2003; Day, 2013; Day et al., 2013). Therapeutic strategies to enhance PQC and induce cardioprotection are of great interest, however a better understanding of the molecular mechanisms regulating cardiomyocyte PQC is needed to facilitate the development of such a novel strategy.

**FIGURE 1:**
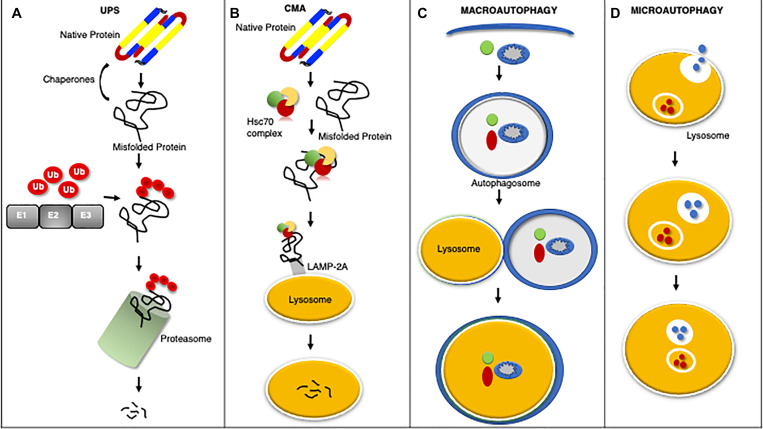
An overview of protein quality control systems. Misfolded proteins may regain native structure with the assistance of chaperones, however if proper re-folding cannot occur the misfolded protein will be catalyzed by the UPS through ubiquitination via a series of enzymatic reactions involving an ubiquitin activating enzyme (E1), ubiquitin conjugating enzyme (E2), and ubiquitin ligase (E3) for degradation by the proteasome **(A)**. Chaperone mediated autophagy (CMA) is a process by which the heat shock cognate 70 (HSC70) complex recognizes and binds select protein targets for internalization and degradation to the lysosome through the lysosome associated membrane protein 2A (LAMP2A) receptor **(B)**. Macroautophagy is the bulk removal of proteins, protein aggregates, and organelles by first forming an autophagosome to surround the cargo followed by merger with the lysosome for degradation **(C)**. Microautophagy is a process by which the lysosome invaginates to bring protein substrates into the lysosome for degradation **(D)**.

Cardiomyocytes utilize elaborate intracellular PQC mechanisms to maintain proteostasis and counter disease progression (Figure 1). Briefly stated the first line of defense against impaired proteostasis are the molecular chaperones which bind to misfolded proteins, whereas degradation of proteins is carried out by the proteasome and lysosome (Wang et al., 2008; Ranek et al., 2018). Protein misfolding can expose a stretch of hydrophobic amino acids to the cytosol. These amino acids are recognized and bound by molecular chaperones to either promote protein re-folding, prevent misfolded proteins from aggregating, and/or facilitate the interaction between the misfolded protein and a ubiquitin ligase (Ranek et al., 2018). The UPS degrades proteins that have been targeted for degradation via a ubiquitin chain, canonically a lysine-48 linked chain (Wang et al., 2008). The UPS consists of the ubiquitination enzymes: an E1 ubiquitin activating enzyme, an E2 ubiquitin conjugating enzyme, and an E3 ubiquitin ligase; along with the barrel shaped structure known as the proteasome which contains the proteolytic activities (Wang et al., 2008, 2011). The lysosome degrades proteins, macromolecules, and whole organelles by processes encompassing autophagy, which involves the delivery of substrates to the lysosome for internalization and degradation (Zheng et al., 2011; Sciarretta et al., 2018b). Autophagy comes in many forms: microautophagy, macroautophagy, chaperone-mediated autophagy (CMA), CASA, and organelle-specific autophagies (e.g., mitophagy) (Henning and Brundel, 2017; Ghosh and Pattison, 2018; Sciarretta et al., 2018b).

A growing area of interest surrounds the role of PTMs in the regulation of PQC pathways. Various PTMs have been reported to promote or hinder cardiomyocyte PQC, including nitrosylation, oxidation, and ubiquitination among others (Wang et al., 2013). By doing so, PTMs have greatly expanded the specificity and capacity of protein degradation during cardiac disease. This review highlights the recent advances of arguably the most established PQC-regulating PTM, phosphorylation. Excellent reviews of the other PTMs have been described elsewhere (Christians and Benjamin, 2012; Scruggs et al., 2012; Wang et al., 2013; Penna et al., 2018; VerPlank and Goldberg, 2018). This review details the recent PQC phosphorylations that have been identified (Supplementary Table 1), especially those with influential roles in cardiac pathophysiology and some ubiquitous signaling pathways, and that can be clinically interrogated.

### Impaired Protein Quality Control (PQC) in Cardiac Disease

**FIGURE 2:**
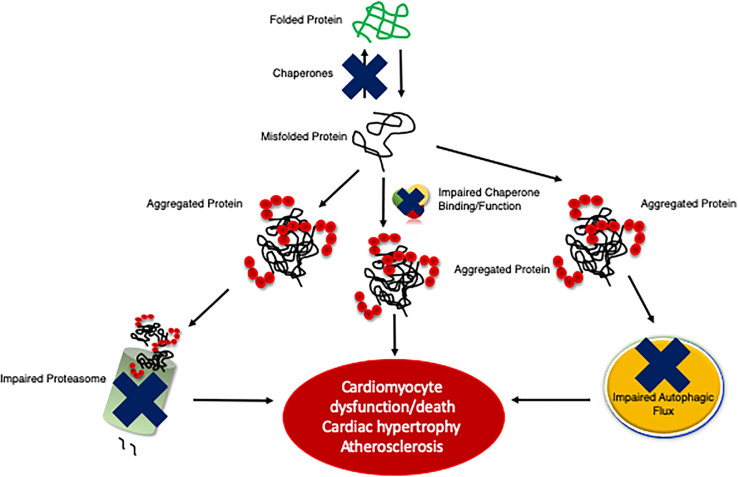
An illustration of the development of cardiac proteinopathy. Cardiomyocytes accumulate ubiquitinated and aggregated proteins as a result of impaired PQC mechanisms. The failure of cardiomyocyte chaperones activity to efficiently re-fold misfolded proteins results in misfolded proteins aggregating, which impairs the proteasome and overwhelms autophagy. Insufficient PQC exacerbates disease pathogeneses (e.g., cardiac hypertrophy and atherosclerosis).

Many forms of cardiac disease are characterized by an accumulation of ubiquitinated proteins and the presence of aberrant protein aggregation in the form of pre-amyloid oligomers (Ranek and Wang, 2009; Parry et al., 2015; Gilda and Gomes, 2017). Indeed, the majority of failing human hearts are characterized by impaired proteostasis, termed cardiac proteinopathies (Su and Wang, 2010; Zheng et al., 2011; Willis and Patterson, 2013). Studies conducted in mice revealed that inadequate cardiomyocyte PQC precedes and exacerbates cardiac pathogenesis (Figure 2) (Wang et al., 2011; Gilda and Gomes, 2017; Henning and Brundel, 2017). The Robbins lab overexpressed a misfolded protein surrogate with a polyglutamine expansion pre-amyloid oligomer (PQ83) in the heart, which impaired PQC leading to reduced cardiac function and ultimately failure (Pattison et al., 2008). Similar results were obtained in another mouse model expressing a mutated αB-crystallin (CryAB^R120G^), a misfolded and aggregation prone protein, in cardiomyocytes alone (Wang et al., 2001, 2003). PQ83 and CryAB^R120G^ mice exemplify a pathological process termed proteotoxicity, which refers to the adverse effects induced by the presence of damaged or misfolded proteins (Sandri and Robbins, 2014). If not corrected, cardiac proteotoxicity will result in apoptosis/necrosis at the cellular level, decreased function at the organ level, and even premature death for the organism (Sandri and Robbins, 2014). Indeed, CryAB^R120G^ mice showed impaired PQC within 3 months of age and heart failure within 6 months of age (Wang et al., 2001, 2003; Kumarapeli and Wang, 2004). Stimulation of protein degradation via genetic upregulation of autophagy rescued the CryAB^R120G^ phenotype (Pattison et al., 2011). Interestingly exercise was also shown to attenuate pre-amyloid deposition, proteotoxicity, and heart failure progression (Maloyan et al., 2007). A recent study by the Wang lab utilized the CryAB^R120G^ as a model of proteinopathy that occurs with HFpEF, a devastating disease currently without a treatment (Sharma and Kass, 2014). CryAB^R120G^ mice were treated with a phosphodiesterase 1 inhibitor, which activated protein kinases A (PKA) and G (PKG), stimulated proteasome activities, and attenuated proteotoxic stress (Zhang et al., 2019). This study demonstrates the potential of targeting protein degradation as a heart failure treatment. Chronic inhibition of any one of the PQC machineries exacerbates cardiac pathogenesis following MI via closure of the left ventricular anterior descending artery or left ventricular PO via transaortic constriction (TAC), whereas the enhancement of cardiomyocyte PQC protects the myocardium facilitating increased proteostasis, cardiac function, and lifespan (Wang and Robbins, 2006; Wang et al., 2008, 2011; Su and Wang, 2010; Ranek et al., 2015).

### Chaperones

The first line of defense against proteotoxicity are chaperones, which are a broad class of proteins that assist with refolding misfolded proteins, facilitating an interaction between misfolded proteins and a ubiquitination enzyme, and preventing proteins from aggregating together (Figure 1) (Willis and Patterson, 2010; Ranek et al., 2018). There are chaperones that are constitutively expressed (e.g., heat shock cognate 70, HSC70) and others, whose expression are induced by a cardiac stress [e.g., heat shock protein 70 (HSP70)] (Wu et al., 1985; Dworniczak and Mirault, 1987). Chaperones can protect cardiomyocytes against proteotoxicity and subsequent cell death during a stressful/pathological condition (Willis and Patterson, 2010; Tarone and Brancaccio, 2014; Penna et al., 2018). Indeed, overexpression of certain chaperones has protected hearts from cardiac disease (Willis and Patterson, 2010; Tarone and Brancaccio, 2014). Multiple studies have reported overexpression of HSP70 to be protective in mouse models of ischemia and ischemia-reperfusion injury (Marber et al., 1995; Plumier et al., 1995; Radford et al., 1996). Another study determined that HSP70 overexpression is protective in periods of brief ischemia where there is myocardial dysfunction but no infarct (Trost et al., 1998). In a pig model of ischemia-reperfusion, HSP90 transfection was protective and reduced the ischemic region (Kupatt et al., 2004). It was suggested that this protective effect was due to HSP90 mediated enhancement of NO formation (Depre et al., 2006). Injection of HSP22 into the swine heart was protective against MI in a NO-dependent manner (Chen et al., 2011), however, it seems that this is only effective acutely, as more recent work shows that chronic overexpression is deleterious (Morin et al., 2019). This discrepancy was due to increased ROS production and oxidative stress that occurs with chronic HSP22 expression that results in cardiac hypertrophy and shortened lifespan (Morin et al., 2019). Another study using a HSP22 overexpression transgenic mouse model reported cardioprotection with increased preconditioning to attenuate MI (Depre et al., 2006). Here, the authors noted a metabolic switch to favor glucose utilization and expression of anti-apoptotic factors over pro-apoptotic factors. These findings were supported by a recent study describing protection against doxorubicin induced cardiotoxicity in HSP22 transgenic mice (Lan et al., 2020).

One of the most recognized protective roles of cardiomyocyte chaperones is to maintain cardiac systole and diastole, through modulation of contractile proteins (Willis et al., 2009, 2010). Impaired cardiac function with increased ventricular stiffness are hallmarks of heart failure. Altered sarcomeric and calcium handling proteins contribute to diastolic left ventricular stiffness. Phospholamban regulates calcium uptake to the sarcoplasmic reticulum via SERCA for cardiomyocyte relaxation to occur. Qian et al. (2011) identified that HSP20 negatively regulates protein phosphatase 1 (PP1) activity via a direct interaction, favoring phosphorylation of phospholamban and increased cardiac function. The small heat shock protein HSPB7 modulates actin thin filament length by binding to monomeric actin and limiting its availability for polymerization (Wu et al., 2017). Zhu et al. (2009) applied single molecule force spectroscopy to determine the contour length and bending rigidity of the N2B-Us of titin and the effect of wild type and mutant R157H (harboring the dilated cardiomyopathy missense mutation), or R120G (desmin-related myopathy mutation) αB-crystallin (CryAB) on the molecular mechanics of the N2B-Us and its flanking Ig domains. CryAB functions as a chaperone that lowers the probability of Ig domain unfolding and the persistence length of the titin N2B-Us spring region. HSP27 or CryAB suppresses cardiomyocyte stiffness caused by stretch and low pH (Kotter et al., 2014). Collectively these studies demonstrate the strong cardioprotective potential of chaperones, however before this potential can be therapeutically leveraged, a greater understanding of the regulatory mechanisms involved in chaperone expression and function is needed (Willis and Patterson, 2010; Tarone and Brancaccio, 2014; Ranek et al., 2018).

#### Chaperones Regulated by Phosphorylation

**FIGURE 3:**
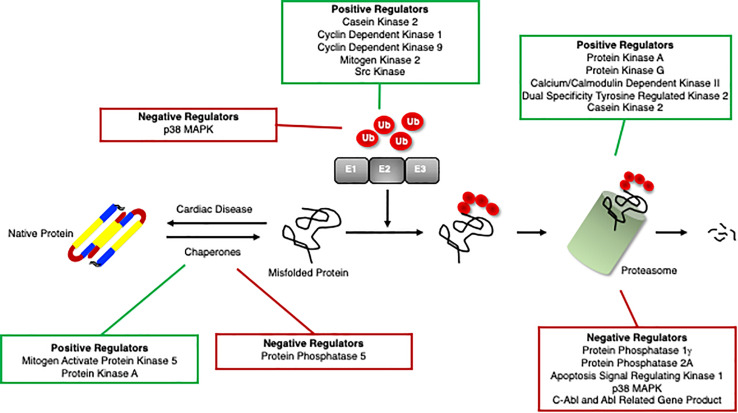
Positive and negative regulators of the ubiquitin proteasome system. Proteins that positively (green box) and negatively (red box) regulate cardiomyocyte chaperones, ubiquitination enzymes, and the proteasome.

Recent studies have identified protein kinases and phosphatases responsible for phosphorylation and dephosphorylation, respectively, of chaperones and the consequent effects on cardiac function (Figure 3). The role of phosphorylation of chaperones and co-chaperones varies depending on the site and even on the temporal regulation of the sites. Some phosphorylation events stabilize the chaperones and their interactions with their client proteins, while others are specific for their client proteins and will only function properly when phosphorylated at the appropriate sites.

The HSP70 family of chaperones support proper protein structure by unfolding and refolding misfolded proteins in an ATP-dependent manner (Sharma et al., 2010; Finka et al., 2015). Phosphorylation has a key role in regulating the function of HSP70s. HSC70 phosphorylation at threonine 38 controls cell cycle progression in a CDK-dependent manner by promoting G1 cyclin binding and subsequent degradation to promote G1/S transition in the cell cycle (Truman et al., 2012). While this study was done in yeast, this phosphorylation site is highly conserved and may play an important role in cardiovascular diseases which typically exhibit high expression of HSC70 (Boehm and Nabel, 2003). The vital role of HSP70s in redox homeostasis is precisely regulated by its phosphorylation at serine 631, which is required for superoxide dismutase 2 (SOD2) import and activation in mitochondria (Zemanovic et al., 2018). Once SOD2 restores the redox balance in mitochondria, HSP70 is dephosphorylated, and both HSP70 and SOD2 are ubiquitinated by CHIP and degraded (Zemanovic et al., 2018). Increased oxidative stress has been associated with the development and pathogenesis of cardiovascular disease. This precise regulation via phosphorylation is pivotal to both maintaining a homeostatic redox state and then binding to proteins damaged by oxidation. Regulatory phosphorylation can occur at the level of the chaperone as well as at the level of the co-chaperone. HSP40, a co-chaperone with HSP70, is phosphorylated by mitogen activated protein kinase 5 (MK5), resulting in enhanced ATP hydrolase function to increase the refolding capacity, thus PQC-maintaining ability, of HSP40/70 (Kostenko et al., 2014).

HSP90 proteins are chaperones with some similar, but different, functions to HSP70s in facilitating protein folding, and also directing proteins for degradation in order to avoid protein aggregation (Hohfeld et al., 2001). The decision-making process for whether to facilitate folding or to direct for degradation is mediated by binding to co-chaperones CHIP and HSP70-HSP90 organizing protein (HOP) (Muller et al., 2013). Phosphorylation is responsible for determining co-chaperone binding and macro-complex formation, such that phosphorylation in the C-termini of HSP70 and HSP90 prevents CHIP binding, but enhances HOP binding, which can assist with protein folding (Muller et al., 2013). Cells in a proliferative state have higher levels of phosphorylated HSP70 and HSP90 and those chaperones are preferentially bound to HOP (Muller et al., 2013). This has major implications in cancer due to the proliferative nature of those cells, but could also impact cardiovascular diseases like atherosclerosis, where vascular smooth muscle cell proliferation is problematic (Bennett et al., 2016). Bachman et al. (2018) demonstrated that the co-chaperone Cdc37 complexes with HSP90 and recruits client kinases, which phosphorylates HSP90 to provide a mechanism for highly specific tuning of the chaperone cycle depending on the client kinase that is recruited. Similarly, dephosphorylation of HSP90 by protein phosphatase 5 was shown to regulate the activity and interacting proteins of HSP90 (Haslbeck et al., 2015; Oberoi et al., 2016). These findings are of particular relevance to the heart as HSP90 is known to be involved in functioning of multiple steroid receptors (Pratt and Toft, 2003; Zhao et al., 2005) ARs play a role in multiple cardiovascular diseases including hypertension, stroke, and atherosclerosis, thus highlighting the therapeutic potential of modulating chaperones (Huang et al., 2016). HSP90 is bound to unliganded AR in the cytosol, however following phosphorylation of HSP90 at threonine 89 by PKA, the AR is released into the cytosol where it binds to HSP27 and migrates into the nucleus to modulates transcription via binding to androgen response elements (AREs) (Dagar et al., 2019).

The cardioprotection elicited by increased molecular chaperones in the heart is well-established (Willis and Patterson, 2010; Tarone and Brancaccio, 2014; Ranek et al., 2018), however, a better understanding of the regulation of chaperones via phosphorylation is desirable for the ability to better fine-tune chaperones’ functions. A mouse model that overexpressed a constitutively phosphorylated HSP20 at serine 16 (previously found to be PKA mediated), promoted fibrotic remodeling and heart failure (Gardner et al., 2019). This came as a surprise, as prior studies suggested that HSP20 phosphorylation at serine 16 was protective against beta-agonist-induced apoptosis in cardiomyocytes (Fan et al., 2004; Qian et al., 2009). A possible explanation for this disparity is the former study examining constitutive HSP20 phosphorylation at serine 16 was done in isolated cardiomyocytes, suggesting that acute phosphorylation of this site is protective, while the effects of chronic HSP20 activation are harmful. Gardner et al. (2019) utilized a ubiquitous knock-in mouse model and attributed this maladaptive response to a pro-fibrotic role of phosphorylated HSP20 that mediates IL-6 activation of cardiac fibroblasts. Another study identified HSP27 phosphorylation at serine 82 as being protective in biomechanically stressed mouse hearts (Collier et al., 2019). Here, they found that actin-binding protein filamin C (FLNC) and HSP27 are upregulated in mouse hearts subjected to left ventricular PO induced by TAC surgery (Collier et al., 2019). It was determined that these proteins interact, with phosphorylation of HSP27 facilitating their binding in a region of FLNC that is mechanosensing, while FLNC localizes to load-bearing sites (Collier et al., 2019). This suggests the mechanism chaperones are recruited in response to force destabilization is to maintain the integrity of the sarcomere and protect the myocardium. HSP27 phosphorylation has also been found to be upregulated in human platelets during ST-elevation post-MI, possibly offering a new measurable stress response for patients with MI (Kraemer et al., 2019).

Another chaperone with significant implications in cardiovascular disease is p97/valosin-containing protein (VCP)/Cdc48, an ATPase involved in a variety of functions to include ERAD, cell cycle progression, autophagy, and DNA repair (Parisi et al., 2018). This ATPase has been demonstrated to play a role in myofibrillar formation, both in limiting normal muscle growth but also contributing to muscle wasting diseases (Piccirillo and Goldberg, 2012). The function of p97 is regulated by phosphorylation at multiple sites (Baek et al., 2013). Phosphorylation of tyrosine 805 disrupts the interaction between p97 and PNGase, attenuating the ERAD pathway, thus PQC (Li et al., 2008). Additional studies found that tyrosine phosphorylation inhibits p97-ER membrane association and transitional ER assembly (Lavoie et al., 2000). Phosphorylation has also been shown to impact the ATPase activity of p97 as well as its association with ubiquitinated proteins (Klein et al., 2005; Mori-Konya et al., 2009). p97 has a role in the DNA damage response, as it is phosphorylated at serine 784 and accumulates at sites of DNA damage, possibly for DNA repair (Livingstone et al., 2005). Taken together, these studies demonstrate the regulatory role of phosphorylation and dephosphorylation on the function of chaperones, presenting many possibilities for targeting these modifications as a therapy.

### Ubiquitination Enzymes

Proteins are targeted for degradation by the proteasome via ubiquitination, a sequential system involving the following enzymes: ubiquitin-activating enzyme (E1), ubiquitin-conjugating enzyme (E2), ubiquitin ligase (E3), and possibly a ubiquitin elongation factor (E4) (Figure 1) (Hershko and Ciechanover, 1998; Goldberg, 2003; Zuo et al., 2020). In humans, there are only 2 E1s that have been identified, and as their name suggests, they are responsible for activating ubiquitin so that it can be conjugated to a protein. This process occurs in an ATP-dependent manner in which the C-terminus of ubiquitin is bound to an E1 cysteine via a thioester bond (Hershko and Ciechanover, 1998; Zuo et al., 2020). The E1 then transfers ubiquitin to one of approximately 40 E2s in humans through another thioester bond (Hershko and Ciechanover, 1998; Zuo et al., 2020). Finally, with an E3 the ubiquitin will be attached to a substrate protein. There are three families of E3s: HECT, RING and RBR, and these families differ slightly in the way that the ubiquitin is transferred to its substrate (Zheng and Shabek, 2017; George et al., 2018). HECT E3s will accept the ubiquitin first from the E2 to form a thioester bond, and will then transfer the ubiquitin to the substrate directly from the E3 (Zuo et al., 2020). RING E3s will bring together the ubiquitin-E2 complex and the substrate and simply mediate the transfer of ubiquitin from the E2 to the substrate (Zheng and Shabek, 2017). RBR ubiquitin ligases act as a hybrid protein of two domains, a canonical RING domain as well as a catalytic cysteine residue similar to the HECT domain (George et al., 2018). The specificity of the UPS is thought to lie within the ubiquitination step (Wang et al., 2008; Ranek et al., 2018). Given the importance of ubiquitination in PQC and degradation, it comes as no surprise that this process and selectivity is further regulated through phosphorylation of these ubiquitination enzymes (Figure 3).

#### Phosphorylations That Regulate Ubiquitination Enzymes

There is limited data into the sites phosphorylated and potential functional change of the two E1s in humans. Stephen et al. (1997) described multiple phosphorylation sites at the N-terminus of E1a. It was previously suggested that E1 could be phosphorylated by PKC, and that E1a serine 4 could be phosphorylated by Cdc2, a kinase that is key in cell cycle regulation (Kong and Chock, 1992; Nagai et al., 1995). In these studies, the function of these phosphorylation sites was not fully described. It was determined that while phosphorylation increases in various states of the cell cycle, the enzymatic activity of E1 was not changed (Stephen et al., 1996). Also, while E1a was found to localize to the nucleus, it was determined that phosphorylation was not required for this localization (Stephen et al., 1997). It is possible that this phosphorylation may increase the rate of nuclear targeting or possibly change the stability of interactions with E2 enzymes, but how phosphorylation may alter these roles is yet to be determined.

Functional alterations of E2 enzymes have been attributed to phosphorylation, albeit the knowledge about them in the heart is limited. A common theme of E2 enzymes and their regulation via phosphorylation is their involvement in the cell cycle. There is evidence of the cell cycle playing a role in cardiovascular diseases such as hypertrophy and atherosclerosis, so these mechanisms of regulation could have interesting therapeutic potential (Bicknell et al., 2003; Ahuja et al., 2007). In one such study of the involvement of an E2 in cell cycle regulation, Cdc34, in yeast was shown to be phosphorylated at serines 203, 222, and 231 in the acidic C-terminal tail domain by casein kinase 2 (CK2) (Sadowski et al., 2007). In an effort to understand the biological importance of these sites, they mutated them to alanine (phospho-null), or a glutamate or aspartate (phospho-mimetics). With the phospho-mutants, they observed differential kinetics through the cell cycle, from G_1_ through S into the G_2_/M phase (Sadowski et al., 2007). The authors were able to associate the cells with faster cell cycle kinetics with an increased rate of Sic1 degradation, and increased Skp, cullin, F-box containing complex (SCF)-mediated ubiquitination of Sic1 (Sadowski et al., 2007). Overall, this was a demonstration that phosphorylation can regulate the catalytic and cell cycle functions of Cdc34. In another study, Shchebet et al. (2012) demonstrated that the ubiquitin-conjugating enzyme E2 A (UBE2A) is directly phosphorylated by CDK9 at serine 120. Knocking down CDK9 reduced UBE2A phosphorylation, which then decreased monoubiquitination of histone H2B and PCNA, suggesting phosphorylation at serine 120 activates UBE2A (Shchebet et al., 2012). This finding is of particular interest to the heart, as a recent study identified monoubiquitination of H2B is a transcriptional regulator that controls expression of cilia genes, where mutations have been implicated in congenital heart disease (Robson et al., 2019). ER stress occurs in various cardiovascular diseases with impaired proteostasis (Ochoa et al., 2018). A study from 2017 identified ubiquitin-conjugating enzyme E2 J1 (UBE2J1) phosphorylation at serine 184 as being important for recovery of ER stress to maintain proteostasis (Elangovan et al., 2017). It was reported that UBE2J1 is phosphorylated at serine 184 by mitogen kinase 2 (MK2) as a mechanism to alleviate ER stress (Menon et al., 2013). Phosphorylated UBE2J1 has higher affinity for the E3 ligase, c-IAP1, and that cells expressing a phospho-null UBE2J1 cannot recover from ER stress (Elangovan et al., 2017). The authors also reported that phosphorylated UBE2J1 is degraded by the proteasome, potentially as a feedback loop (Elangovan et al., 2017).

An E3 ligase that is implicated in multiple diseases, including cardiovascular and neurodegenerative diseases, is Parkin, which is known to be regulated by phosphorylation (Wang et al., 2018). One study demonstrates phosphorylation negatively regulates Parkin’s protective function (Chen et al., 2018). Here, they use a mutant α-synuclein model that, when overexpressed, activates p38 MAPK, which then directly phosphorylates Parkin at serine 131. This disrupts PTEN-induced kinase 1 (PINK1)-Parkin mediated mitophagy and exacerbates the mitochondrial impairment induced by α-synuclein accumulation (Chen et al., 2018). A few studies have highlighted the importance of Parkin in the heart by performing knockout experiments. One such experiment knocked out Parkin in flies and observed accumulation of dysfunctional mitochondria and dilated cardiomyopathy (Bhandari et al., 2014). This phenotype could be rescued with cardiomyocyte-specific Parkin expression (Bhandari et al., 2014). Another study found that in response to MI the parkin knockout mice fared worse than WT mice in terms of survival and infarct size (Kubli et al., 2013). Collectively these studies suggest determining the kinases capable of phosphorylating Parkin in the heart and the role of this PTM during cardiac pathogenesis could provide a novel therapeutic avenue.

The neural precursor cell expressed developmentally down-regulated protein 4 (NEDD4) is a HECT domain ubiquitin ligase that is required for heart development, turnover of potassium and sodium channels, and maintenance of proteostasis (Fouladkou et al., 2010; Gilda and Gomes, 2017). FGFR1 activation results in tyrosine phosphorylation of four key sites (43, 365, 366, and 585) on NEDD4 (Persaud et al., 2014). Site-directed mutagenesis of these four tyrosines revealed that expression of a phospho-mimetic results in constitutive activation of NEDD4 ubiquitin ligase activity, while phospho-null NEDD4 decreased its ubiquitin ligase activity (Persaud et al., 2014). FGF signaling is known to play a pathophysiological role in the heart as evidenced by worsened cardiac hypertrophy in isoproterenol stimulated hearts of FGF2 transgenic mice (House et al., 2010; Itoh and Ohta, 2013). FGF2 KO mice were protected against cardiac hypertrophy and fibrosis following isoproterenol stimulation (Itoh and Ohta, 2013). FGF2 is mostly expressed in non-myocytes within the heart, suggesting cell-to-cell communication as the mode of cardiomyocyte regulation (Itoh and Ohta, 2013). Considering the requirement of NEDD4 in cardiac development, the presence of FGF in all cell types of the heart, and its role in heart disease, this signaling axis presents an interesting therapeutic target.

SUMOylation and neddylation are other PTMs that are analogous to ubiquitination, utilize a similar E1, E2, and E3 mechanism of conjugating either SUMO or NEDD8 to proteins, and are known to be involved in many different biological processes (Wilkinson and Henley, 2010; Zhou et al., 2019). There is evidence of the modifiers SUMO-1 and NEDD8 themselves being phosphorylated, but the functional implications are still unknown (Matic et al., 2008; Enchev et al., 2015). We are only beginning to understand the regulation of neddylation E1 (NEDD8-activating enzyme NAE) and E2s (UBE2M and UBE2F) by phosphorylation. Interestingly, the E3s that have been identified for neddylation can also function as ubiquitination E3s (Enchev et al., 2015). Furthermore, phosphorylation is responsible for converting an E3 from ubiquitin conjugating to NEDD8 conjugating (Batuello et al., 2015). Under growth factor stimulation, src kinase phosphorylates Mdm2 at tyrosines 281 and 302, recruits the NEDD8 E2 (Ubc12) and Mdm2, to neddylate p53 in human cancer cell lines (Batuello et al., 2015). This has interesting implications if this mechanism applies to other E3s. While phosphorylations have been identified on SUMOylation E1 and E2, the function of these modifications is not known (Tomanov et al., 2018). However, there are multiple studies that have found that phosphorylation modifies the function of SUMO E3s. One such study identified Ubc9 as being phosphorylated by CDK1 at serine 71 to enhance SUMOylation activity *in vitro* (Su et al., 2012). Another study in HEK 293T cells elucidates a feedback system whereby DNA-damage induced homeodomain interacting protein kinase 2 (HIPK2) phosphorylates proprotein convertase (Pc2) at threonine 495, which in turn controls Pc2 SUMOylation of HIPK2 in order to enhance its ability to mediate transcriptional repression (Roscic et al., 2006). Additional studies are needed to determine the potential role phosphorylation of the neddylation and SUMOylation proteins may have in cardiac disease pathogenesis.

### The Proteasome

The UPS plays a key role in eukaryotic cells by degrading damaged, defective, and non-functional proteins (Figure 1) (Jentsch, 1992; Schwartz and Ciechanover, 1992; Ciechanover, 1994). This pathway represents non-lysosomal degradation of cellular proteins and plays a critical role in PQC (Goldberg, 2003; Divald and Powell, 2006). Proteins are destined to proteasome-mediated degradation by the formation of a ubiquitin chain on the target protein. Ubiquitinated proteins are fed through the proteasome core complex for degradation *via* an ATP driven process (Collins and Goldberg, 2017). The mammalian proteasome is a highly sophisticated multi subunit protease complex composed of the 20S catalytic core particle, within which proteins are degraded, and two 19S regulatory cap particles which locates and binds to ubiquitinated substrate (Budenholzer et al., 2017; Collins and Goldberg, 2017). The 20S particle is formed by two copies of 14 different subunits (α1–α7 and β1–β7) stacked in heptameric rings (Groll et al., 1997; Budenholzer et al., 2017). The active sites of the catalytic subunits (β1, β2, and β5) lines the central lumen of a chamber gated by an α subunit at its either end (Groll et al., 1997; Budenholzer et al., 2017). The proteasome activator 700 (PA700) or 19S cap has two distinct sub-components (Lasker et al., 2012). The base consists of six Rpt (1→6) subunits that have constitutive ATPase activity, plus two non-ATPase subunits, Rpn1 and Rpn2; and the lid structure contains 10 Rpn (3→12) subunits (Lasker et al., 2012; Budenholzer et al., 2017). The 19S subunit is responsible for binding and unfolding ubiquitinated proteins and feeding the protein substrate to the 20S proteasome for catalysis (Wang et al., 2008).

Several studies have shown a central role of the UPS in cardiovascular physiology and pathophysiology (Razeghi et al., 2006; Drews et al., 2010; Powell and Divald, 2010; Predmore et al., 2010; Zu et al., 2010; Li et al., 2011). Cardiac proteasomes exhibit distinct functional and subcellular distribution (Gomes et al., 2006, 2009; Drews et al., 2007; Powell et al., 2008; Dahlmann, 2016). A substantial number of studies have shown impaired proteasomal function contributes to heart disease (Su and Wang, 2010; Willis et al., 2010; Wang et al., 2011; Day, 2013), which is supported by findings of insufficient UPS activity in human end stage heart failure (Hein et al., 2003; Weekes et al., 2003; Predmore et al., 2010). Proteasome dysfunction has been reported in hypertrophic, ischemic, atrophic, desmin-related, and diabetic cardiomyopathies (Razeghi et al., 2006; Drews et al., 2010; Powell and Divald, 2010; Predmore et al., 2010; Zu et al., 2010; Li et al., 2011). Arguably the most well-studied cardiac proteinopathy mouse model is DRC caused by mutations in desmin, αB-crystallin (CryAB), and other related genes (Wang and Robbins, 2006). The DRC model features aberrant protein aggregates in myocytes, reduced cardiac function, and a shortened lifespan (Wang et al., 2001, 2003; Pattison et al., 2011). These hearts also have PFI, which was postulated to contribute further to the impaired proteostasis and the pathogenesis of DRC (Wang and Robbins, 2006; Su and Wang, 2010; Wang et al., 2011). Studies enhancing proteasome activity in DRC have supported this notion (Li et al., 2011; Ranek et al., 2013). PFI has also been observed in mouse hearts subjected to ischemia/reperfusion (I/R) injury and transaortic constriction (TAC)-induced cardiac PO (Wang et al., 2011). Expression of a catalytically inactive proteasome beta5 subunit negates the chymotrypsin-like activity, but not the caspase- or trypsin-like activities, and worsened cardiac PQC and function following I/R or TAC (Ranek et al., 2015).

Methods to enhance proteasome activities have been shown to be protective. Post-translational modifications are key regulatory mechanisms for proteasome function. More than 300 phosphorylation sites in proteasome subunits have been detected that can regulate protein stability, abundance, assembly, subcellular localization, substrate recognition and enzymatic activity of proteasome subunits (Benedict and Clawson, 1996; Satoh et al., 2001; Bose et al., 2004; Zong et al., 2006; Bingol et al., 2010; Guo et al., 2011, 2016; Djakovic et al., 2012; Sledz et al., 2013; Yuan et al., 2013; Li et al., 2015; Lokireddy et al., 2015; Wani et al., 2016). In humans, 20S cap and the 19S base subunits are more frequently phosphorylated than the 19S lid. Many kinases have been demonstrated to regulate proteasome activity in general and in heart tissues (Figure 3) (Powell and Divald, 2010; Sledz et al., 2013; Wang et al., 2013; Collins and Goldberg, 2017; VerPlank and Goldberg, 2018).

### Phosphorylations Regulating the Proteasome

#### Protein Kinase A (PKA)

Protein kinase A was the first kinase identified to phosphorylate proteasome subunits and stimulate proteasome activities *in vitro* as well as *in vivo* (Zong et al., 2006, 2008; Asai et al., 2009; Gomes et al., 2009; Drews et al., 2010). A series of studies using bovine pituitaries, and later from murine hearts, identified PKA in a complex with endogenous 20S proteasomes (Pereira and Wilk, 1990; Zong et al., 2006; Drews et al., 2010). PKA phosphorylation of the proteasome has been detected on serine residues of the α 1-, α 2-, α 3-, β 2-, β 3-, and β7-subunits, and the threonine residues of the α 3-, β 3-, and β7-subunits of the 20S proteasome (Zong et al., 2006). Additionally studies demonstrated enhancement of proteasome activity via PKA stimulation. The addition of cAMP stimulates PKA activation and its interaction with the proteasome, which resulted in increased phosphorylation of 19S cap subunit Rpn6 at Ser14 (Lokireddy et al., 2015). Pathare and others have shown that raising cAMP levels increases the amount of double-capped 26S proteasomes, indicating increased stabilization of the proteasome complexes (Pathare et al., 2012; VerPlank et al., 2019). Rpn6 serine 14 phosphorylation has been detected *in vivo* in response to fasting, intense exercise, and hormonal cues which increases intracellular cAMP and PKA activity (VerPlank et al., 2019). PKA has also been reported to activate the proteasome via phosphorylation of the ATPase subunit Rpt6 (Zhang et al., 2007). Recombinant PKA directly phosphorylates Rpt6 at serine 120 *in vitro*, and expression of a Rpt6 phospho-silenced Ser120Ala mutation reduced the proteasome activity (Zhang et al., 2007). Phosphorylation of the 19S cap Rpt6 ATPase subunit initiates assembly of 26S proteasome, by stimulating the association of the 19S particle with the 20S proteasome (Satoh et al., 2001). Subsequent studies have reproduced the changes in proteasome activity by modulating PKA activity, however, they have failed to confirm phosphorylation of several proteasome subunit sites (VerPlank and Goldberg, 2018). There are various explanations for this discrepancy: a kinase may modify additional targets in an artificial setting, *in vitro* but not *in vivo*, only in specific cell types, and that kinases and phosphatases may co-purify with the proteasome which can add or remove phosphate groups during isolation (Guo et al., 2017; VerPlank and Goldberg, 2018). Nonetheless, these studies demonstrate PKA is a critical regulator of proteasome assembly and activity during physiological and pathological conditions.

#### Protein Phosphatases 1γ (PP1γ) and 2A (PP2A)

Dephosphorylation via protein phosphatases of the proteasome negatively regulates the activity of the proteasome. Protein phosphatase 1γ (PP1γ) was postulated to reverse the effect of PKA phosphorylation on 20S proteasome activity (Zhang et al., 2007). Protein phosphatase 2A (PP2A) has also been reported to interact with and dephosphorylate the native 20S subunits in cardiac proteasome (Zong et al., 2006). PP2A reduced serine phosphorylation on α1 and β7, and threonine phosphorylation on α1, were linked to suppressed proteasome activity. Echoing these results, pharmacological inhibition of PP2A by OA conferred increased proteasome activity (Gomes et al., 2006; Zong et al., 2006). These data demonstrate the regulatory role of these phosphatases, however whether they can be targeted during disease pathogenesis to enhance proteostasis remains to be determined.

#### Calcium/Calmodulin-Dependent Protein Kinase II (CaMKII)

A rise of intracellular calcium stimulates proteasome activity by way of calcium/calmodulin dependent protein kinase (CaMKII) phosphorylation of the proteasome (Djakovic et al., 2009). Purified CaMKIIα phosphorylated Rpt6 at serine 120 resulting in increased 26S proteasome activity in both neurons and HEK 293T cells (Djakovic et al., 2009). Phosphorylation of Rpt6 at serine 120 was found to be important for neuronal plasticity (Jarome et al., 2013). Activated CaMKIIα interacts with proteasome complex which is essential for proteasome redistribution to dendritic spines for degradation of polyubiquitinated proteins (Bingol et al., 2010; Djakovic et al., 2012; Hamilton et al., 2012). Interestingly in the presence of calcium, calmodulin binds to several proteasome subunits and proteasome-interacting proteins, which may alter 26S function allosterically without involvement of a kinase (Shen et al., 2005; Djakovic et al., 2009, 2012; Bingol et al., 2010). These findings are of particular relevance to the cardiac field as both intracellular calcium and activation of CaMKII are known to worsen cardiac disease pathogenesis (Anderson et al., 2011), although CaMKII regulation of the cardiac proteasomes and proteostasis in a diseased state is yet to be explored.

#### Dual-Specificity Tyrosine-Regulated Kinase 2 (DYRK2)

The proteasome regulates many cellular processes, including cell cycle progression where accumulating evidence suggests a pivotal role for proteasome phosphorylation. Guo and colleagues reported proteasomes purified from S phase of the cell cycle contained increased phosphorylated Rpt3 (of the 19S cap) at threonine 25 and remained high through G2 and M phases (Guo et al., 2016). Utilizing an unbiased screen of human kinases, they found that dual-specificity tyrosine-regulated kinase 2 (DYRK2) could catalyze Rpt3 phosphorylation at threonine 25 to promote cell proliferation (Guo et al., 2016). Prevention of this phosphorylation through expression of a phospho-null threonine to valine (Thr25Val) mutation in human breast cancer cells blocked the stimulation of the proteasomes by DYRK2, thus slowing the degradation of essential cell cycle regulatory factors like cyclin-dependent kinase inhibitor 1B (p27^Kip1^) and cyclin-dependent kinase inhibitor 1 (p21^Cip1^) (Guo et al., 2016). DYRK2 phosphorylation of Rpt3 threonine 25 increased substrate-stimulated ATP-hydrolysis, without changing basal ATPase activity, indicating that the modification promotes substrate translocation and degradation (Guo et al., 2016). The role of DYRK2 on the proteasome remains to be explored in striated muscle and as a potential therapeutic target for cardiac proteotoxicities.

#### Apoptosis Signal-Regulating Kinase 1 (ASK1)

Proteasome phosphorylation does not always result in increased activity. The ASK1 is a member of the mitogen-activated protein kinase kinase kinase (MKK) family and is activated by several cytotoxic stressors and apoptotic stimuli (Nishida et al., 2017). ASK1 negatively regulates the 26S proteasome under oxidative stress or apoptosis through phosphorylation of the 19S cap subunit Rpt5, however it remains unclear which sites of Rpt5 are modified by ASK1 (Um et al., 2010). Proteasome regulation by ASK1 could be an underlying mechanism of its role in cardiac pathogenesis. Deletion of ASK1 protected hearts from MI and PO as evidenced by decreased fibrotic remodeling and apoptosis with improved cardiac function (Yamaguchi et al., 2003). Similar results were obtained with pharmacological inhibition of ASK1 in a model of pulmonary arterial hypertension (Budas et al., 2018). Together these studies suggest the cardioprotective effects of ASK1 inhibition could be mediated by enhanced proteasome activities.

#### P38 Mitogen Activated Protein Kinase (MAPK)

The Rpn2 subunit of the 19S cap acts as a scaffold for other proteasome subunits and with other 19S subunits, regulates its gate opening of the 26S proteasome into the 20S core (Chen et al., 2016; Finley et al., 2016; Schweitzer et al., 2016). Phosphorylation of Rpn2 at threonine 273 by p38 MAPK suppresses proteasome function (Lee et al., 2010). It has been speculated that this phosphorylation of Rpn2 causes a conformational change, affecting the accessibility of substrates to the 20S core (Kors et al., 2019). Lee et al. (2010) reported purified 26S proteasomes from HeLa cells expressing activated p38 MAPK, had reduced proteolytic activities. The authors also showed that overexpression of an active mutant of p38 MAPK caused the accumulation of proteins that normally undergo rapid proteasomal degradation by ubiquitin-dependent and -independent pathways. p38 MAPK mediated phosphorylation of Rpn2 at threonine 273 is stimulated by sorbitol and NaCl-induced osmotic stress (Lee et al., 2010). Small molecule inhibitors of p38 MAPK and siRNA-mediated knockdown of its α-isoform stimulated proteasome peptidase activity and intracellular degradation of α-synuclein in neurons (Leestemaker et al., 2017). Of note, Tsai et al. (2015) has shown that another MAPK, ERK 2, could also phosphorylate the 19S cap subunit Rpn2 at threonine 273 *in vitro.* While p38 is known to be involved in that pathogenesis of cardiac disease (Yokota and Wang, 2016) its effect on the proteasome in the heart remains to be elucidated.

#### Casein Kinase II (CK2)

Casein kinase II (CK2) is a pivotal regulator of the proteasome and is dysregulated in heart failure. CK2 is co-purified with the 20S proteasome and has been demonstrated to phosphorylate the α7 subunit at serines 243 and 250, which is associated with stabilization of the 26S proteasome (Ludemann et al., 1993; Castano et al., 1996; Bose et al., 2004). The phosphorylation of serines is important for the interaction between the 19S cap proteins with the 20S regulatory proteins of proteasome complex (Bose et al., 2004). Interestingly, phosphorylation of the 20S proteasome α7 subunit at serine 250 is found to be significantly less in the tissues from end stage heart failure patients, which is consistent with the diminished activity of the UPS in these patients (Hein et al., 2003; Weekes et al., 2003; Predmore et al., 2010). However, CK2 expression was found to be higher by almost 70% (*P* = 0.019) in failing hearts (Day et al., 2013). The possible explanation could be due to by increased expression of PP1 and PP2A in myocardium of such patients which can dephosphorylate this site (Neumann et al., 1997; Zong et al., 2006; Hamdani et al., 2010), or increased oxidation and carbonylation of CK2 resulting in diminished activity of the kinase (Murtaza et al., 2008).

#### C-Abl and Abl-Related Gene Product (ARG)

As key regulators of cardiac growth and development, C-Abl and ARG (Abl-related gene product) are multi-functional tyrosine kinases, which also directly phosphorylate and regulate the proteasome (Liu et al., 2006; Qiu et al., 2010; Li et al., 2016). Phosphorylation of the α4-subunit at tyrosine 153 by c-Abl led to the inhibition of the 20S and 26S proteasome and decreased the degradation of ubiquitinated short-lived proteins in mouse and human cells (Liu et al., 2006; Li et al., 2015). Activation of c-Abl by H_2_O_2_ or γ-irradiation increased its interaction with the α4-subunit and inhibited proteasome function (Liu et al., 2006). Expression of a phospho-null α4 subunit tyrosine 153 mutant in HEK 293T cells resulted in downregulation of several cell cycle regulatory proteins and G1/S cell cycle arrest, indicating the prominent role of c-Abl/ARG in cell cycle control (Liu et al., 2006). In a separate study, it was shown that phosphorylation of α4 at a different site, tyrosine 106, by c-Abl/ARG regulated the turnover of the proteasome subunit itself (Li et al., 2015). These interesting studies could provide insight into the role of the proteasome in cardiac growth and development as well as its regulation.

#### Protein Kinase G (PKG)

Activation of PKG protects the myocardium against pressure-overload and ischemia-reperfusion (Takimoto et al., 2005; Nagayama et al., 2009; Zhang and Kass, 2011; Sasaki et al., 2014; Kokkonen and Kass, 2017; Ranek et al., 2019). There are also a plethora of therapeutic strategies capable of activating PKG in the heart (Dunkerly-Eyring and Kass, 2019). PKG activators have shown such immense promise in pre-clinical studies that many clinical trials were initiated with various PKG activation strategies to treat human heart failure (Dunkerly-Eyring and Kass, 2019; Pinilla-Vera et al., 2019; Oeing et al., 2020a). Stimulation of PKG via pharmacological activation of the muscarinic 2 receptor or inhibition of phosphodiesterase 5A, positively regulates cardiomyocyte proteasomal proteolytic activity (Ranek et al., 2013, 2014). Stimulation of PKG enhanced proteasome-mediated degradation of a misfolded protein substrate (a modified GFP harboring a degron sequence) and a bona fide misfolded protein CryAB^R120G^ (Ranek et al., 2013). The mechanism of action involved increased phosphorylation of the 20S subunit β5 and the 19S ATPase subunit Rpt6 upon PKG activation in cardiomyocytes (Ranek et al., 2013). The residues phosphorylated by PKG were not identified in this study. It is noteworthy that proteasome-mediated degradation of misfolded proteins was enhanced but not that of normal (properly folded) proteins with PKG stimulation, increasing the safety profile as a potential therapy (Ranek et al., 2013). Accordingly, PKG activators are safe and well-tolerated in human patients (Dunkerly-Eyring and Kass, 2019; Pinilla-Vera et al., 2019). A recent study that utilized human HFpEF myocardium biopsies found that *in vitro* treatment with an guanylyl cyclase 1 (GC-1) activator (to activate PKG) resulted in lower levels of inflammatory cytokines and oxidative stress (Kolijn et al., 2020). With oxidative stress being a known cause of protein damage and activation of the protein damage response, this reduction in oxidative stress can alleviate the burden on the PQC systems such as the proteasomal degradation pathway (Aiken et al., 2011). Taken together, the combination of promising pre-clinical studies, human clinical trials, and availability of pharmacological agents make activators of PKG an attractive therapeutic strategy for cardiac proteinopathies.

## Autophagy

Autophagy is an intracellular self-degradative process responsible for the removal of misfolded proteins damaged organelles and by the lysosome. Cardiomyocytes utilize many forms of autophagy which differ by the method of degradation and the substrates targeted for degradation (Figure 1) (Sciarretta et al., 2018b). Macroautophagy begins with the formation of a double-membrane vesicle called phagophore, through a series of highly coordinated steps termed induction, nucleation and elongation, which engulf malformed proteins or whole organelles in the cytoplasm to form autophagosomes (Klionsky, 2005; Suzuki and Ohsumi, 2007; Levine and Kroemer, 2008). A fully formed autophagosome will then fuse with the acidic compartment of lysosome for the degradation of its cargo molecules (Sciarretta et al., 2018b). The non-selective lysosomal degradative process called microautophagy involves direct engulfment of cytoplasmic cargo at a boundary membrane by autophagic tubes, which mediate both invagination and vesicle scission into the lumen (Su et al., 2011). There are two types of autophagy in mammalian cells which involves degradation of cellular components via a chaperone. Chaperone-mediated autophagy (CMA) is a selective autophagic process mediated by a HSC70 chaperone complex, wherein the proteins for degradation are selected based on a consensus sequence of amino acid sequence present on the surface of protein (Kaushik and Cuervo, 2018; Juste and Cuervo, 2019). On the other hand, CASA is a highly selective autophagy of misfolded proteins following a chaperone-mediated formation of protein aggregates that are targeted to form autophagosomes (Ulbricht et al., 2015). The vast majority of studies into the role of autophagy in the heart and disease pathogenesis have investigated macroautophagy. This section describes our current knowledge into the roles and regulations of autophagy by phosphorylation in cardiac disease, primarily discussing macroautophagy due to the limited understanding of the regulations of other forms of autophagy.

**FIGURE 4:**
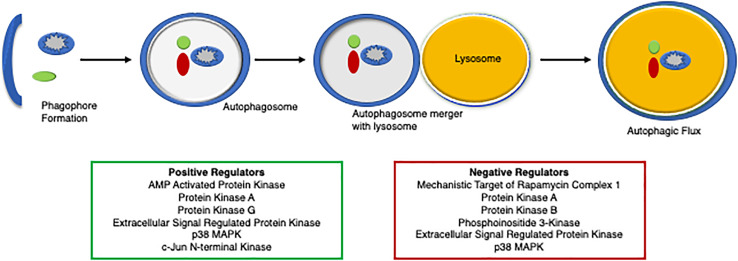
A diagram of the regulators of autophagy. An overview of protein degradation via autophagy and its regulators, both positively (green box) and negatively (red box).

Evidence of autophagic impairment in human heart disease was first reported in tissue samples from patients with dilated cardiomyopathy (Shimomura et al., 2001). Autophagy is repressed in failing hearts as evidenced by the accumulation of numerous autophagic vacuoles containing cytoplasmic material and organelles to be degraded within the degenerated cardiomyocytes, which is thought to contribute to pathological remodeling and heart failure (Shimomura et al., 2001). Reduced autophagy is also implicated in cardiovascular decline and aging (Eisenberg et al., 2016; Shirakabe et al., 2016). Autophagy protects the heart during ischemia and starvation by supplying substrates for maintaining cellular bioenergetics (Matsui et al., 2007; Hariharan et al., 2010; Zhai et al., 2011; Sciarretta et al., 2012a; Kubli et al., 2013). Activation of autophagy has protected the myocardium against pathological cardiac hypertrophy and proteotoxicity (Gao et al., 2006; Kuzman et al., 2007; Flynn et al., 2013; Dai et al., 2014). These seminal studies utilized a genetic overexpression of an autophagy protein to stimulate autophagy, while recent studies have identified critical PTMs that regulate autophagy. More than 300 PTMs have been characterized for various autophagic proteins that influence their structure and function (Xie et al., 2015; Botti-Millet et al., 2016). Here, we discuss the role of phosphorylation as a critical PTM and its functional relevance in fine-tuning the autophagic process (Figure 4).

### Phosphorylations Regulating Autophagic Flux

#### Mechanistic Target of Rapamycin Complex 1 (mTORC1)

Arguably the most well-defined regulator of macroautophagy is the mechanistic (mammalian) target of rapamycin complex 1 (mTORC1), an atypical serine/threonine kinase that also regulates cellular metabolism and bioenergetics. Pro-growth signals and a nutrient-rich environment are associated with mTORC1 activation which negatively regulate macroautophagy. Cardiac PO, obesity, and metabolic syndrome are associated with hyperactivation of mTORC1 (Choi et al., 2012; Li et al., 2012; Ramos et al., 2012; Guo et al., 2013; Pires et al., 2017). Indeed, the hearts from pressure overloaded mice, *ob/ob* obese mice, mice with hypertrophic cardiomyopathies, and hearts from a swine model of metabolic syndrome exhibit mTORC1 activation, depressed autophagic flux, and cardiac functional impairment, all of which were rescued by inhibiting mTORC1 via rapamycin administration (Choi et al., 2012; Li et al., 2012; Ramos et al., 2012; Guo et al., 2013; Pires et al., 2017).

Macroautophagy is influenced by mTORC1 by various methods and pathways. Autophagosome formation is reduced through mTORC1 phosphorylation of autophagy related (Atg) 13, which decreases its affinity for Atg1 (also known as Unc-51 like autophagy activating protein kinase, Ulk1), resulting in the dissociation of the Atg13-Atg1-FIP200 complex (Papinski and Kraft, 2016). Direct phosphorylation of Atg1 (Ulk1) by mTORC1 is another strategy by which mTORC1 suppresses autophagosome formation (Sciarretta et al., 2012b, 2018a). Recently protein phosphatase 2A (PP2A) was identified as a phosphatase for Ulk1 at serine 637, which counteracts the autophagy inhibiting action of mTORC1 (Wong et al., 2015). The Ambra1 is a positive regulator of autophagy that is inhibited by mTORC1 phosphorylation at serine 52 (Nazio et al., 2013). The role of Ambra1 is not well-defined in cardiac disease, however it has been associated with the pathogenesis of Alzheimer’s Disease, aging, and tumor growth (Cianfanelli et al., 2015). Cardiomyocyte macroautophagy is tightly linked to the protein expression levels of Atg7 (Bhuiyan et al., 2013), which is negatively regulated by mTORC1 by reducing its expression level (Ramos et al., 2012). Overexpression of Atg7 enhances macroautophagy to protect the myocardium from cardiac proteinopathy, as evidenced by improved cardiac function and extended lifespan (Pattison et al., 2011). The TFEB is a master regulator, driving genes encoding autophagic proteins like ultraviolet radiation resistance associated gene (*UVRAG)*, WD repeat domain phosphoinositide-interacting protein (*WIPI)*, microtubule-associated protein 1 light chain 3 beta (*MAPLC3B), sequestosome 1 (SQSTM1), vacuolar protein sorting-associated protein 11 (VPS11), VPS18*, and *Atg9B* (Settembre et al., 2011). mTORC1 negatively regulates TFEB through phosphorylation at serines 142 and 211, which prevents the nuclear localization and transcriptional activity of TFEB and thereby decreasing macroautophagy (Napolitano et al., 2018). Buss et al. (2009) and others have shown that hyperactivated mTORC1 in the heart blunts autophagy and profoundly increases pathological cardiac remodeling in response to chronic ischemic injury, and can be reversed by direct mTORC1 inhibition with everolimus. Taneike et al. (2016) have shown that knocking out TSC2 (tuberin), an upstream negative regulator of mTORC1, results in constitutive stimulation of mTORC1 in the heart and subsequent cardiac chamber dilatation and dysfunction associated with an impairment of autophagic flux, which were reversed by pharmacological inhibition of mTORC1. GSK-3α knockout mice display premature death and age-related cardiac abnormalities, such as hypertrophy and sarcomere disruption, as a result of mTORC1 activation and autophagy inhibition (Zhou et al., 2013). Inactivation of Ras homolog enriched in brain (Rheb) (a positive mTORC1 regulator) protects cardiomyocytes during energy deprivation via depressing mTORC1 hyperactivation resulting in activation of autophagy, reduction of energy expenditure and attenuation of ER stress in high fat diet-induced metabolic syndrome (Sciarretta et al., 2012a). Similarly, long-term caloric restriction attenuates the LV diastolic dysfunction of the aged rat heart by reducing mTORC1 activity and enhancing autophagic flux (Shinmura et al., 2011). These studies demonstrate the regulatory capacity of mTORC1 on macroautophagy and cardioprotective effects that mTORC1 inhibition may have, however targeting mTORC1 as a treatment regimen is complex.

Considering mTORC1 is a critical regulator of many physiological processess, the role of mTORC1 and autophagy during cardiac development and pathogenesis is nuanced. Riehle et al. (2013) has shown that insulin/IGF-1 signaling through the insulin receptor substrate 1 proteins are vital to perinatal development of the heart. Reduced IRS signaling prevents the physiological activation of mTORC1 to suppress autophagy resulting in unrestrained autophagy in cardiomyocytes, which contributes to myocyte loss, heart failure, and premature death (Riehle et al., 2013). Knocking out raptor, the scaffolding protein of mTORC1, inhibits mTORC1 and leads to dilated cardiomyopathy (Shende et al., 2011). Furthermore, chronic mTORC1 inhibition with rapamycin is associated with immunosuppression (Thomson et al., 2009). Collectively these studies highlight the risk and reward that comes with broad mTORC1 inhibition. Inhibitors of mTORC1 that inhibit the pathological but not physiological roles of mTORC1 are of great interest.

#### AMP-Activated Protein Kinase (AMPK)

AMP-activated protein kinase is an intracellular metabolic energy sensor that functions to maintain homeostatic levels of ATP. Stimulation of AMPK results in inhibition of anabolic pathways consuming ATP and activation of catabolic pathways generating ATP. Cardiac insults such as myocardial ischemia, hypertrophy, and heart failure are associated with reduced intracellular ATP and subsequent activation of AMPK as a compensation to increase autophagy (Kubli and Gustafsson, 2014). Many studies have demonstrated that AMPK inhibits pathological cardiac hypertrophy by activating autophagy (Li et al., 2014). A study by Xie et al. (2011) suggested activation of AMPK and subsequent increase in cardiac autophagy reduced myocyte apoptosis in diabetic mouse hearts. AMPK activity was reduced in a mouse model of severe early-onset type-1 diabetes, which subsequently suppressed autophagy and increased cardiomyocyte apoptosis. Restoration of AMPK activity with metformin prevented the development of cardiomyopathy (Xie et al., 2011).

AMPK can stimulate autophagy through multiple mechanisms. AMPK activates TSC2 (an upstream negative of mTORC1) though phosphorylation at serine 1387 to inhibit mTORC1 (Inoki et al., 2003). AMPK also directly phosphorylates raptor (mTORC1 scaffold protein) at serines 722 and 792 to reduce mTORC1 activity by inducing 14-3-3 association (Gwinn et al., 2008). Atg1 (Ulk1) is positively and negatively regulated by phosphorylation. mTORC1-mediated phosphorylation of Ulk1 at serine 757 disrupts the Ulk1-AMPK interaction by inducing an unstructured and intrinsically disordered region in the protein (Kim J. et al., 2011; Shang et al., 2011; Khan and Kumar, 2012). Kim J. et al. (2011) have shown that under glucose starvation, AMPK promotes autophagy by phosphorylating Ulk1 at serines 317 and 777, thus activating Ulk1. Under conditions of mitochondrial stress, AMPK phosphorylates Ulk1 at serine 555 to enhance mitophagy by promoting translocation of Ulk1 to mitochondria. Tian et al. (2015) created a phosho-silenced Ulk1 by mutating serine 555, which blocked AMPK phosphorylation and subsequent mitophagy, demonstrating the necessity for this site to be targeted. AMPK phosphorylates beclin1 at serines 91 and 94 in response to glucose starvation, and serines 93 and 14 following exposure to ethanol (Kim et al., 2013; Hong-Brown et al., 2017). AMPK mediated phosphorylation of beclin1 at threonine 388 enhances the association of beclin1 with the VPS34-ATG14-VPS15 complex to stimulate autophagy and also reduces beclin1-Bcl2 complex formation (Zhang et al., 2016), which is known to inhibit autophagy and induce apoptosis. A beclin1^*T388A*^ phospho-silenced mutant suppresses autophagy by inhibiting its interaction with ATG14 (Zhang et al., 2016). The role that phosphorylation of these sites on beclin1 has during cardiac disease remains to be explored but may hold potential as multiple studies demonstrated the important role of becin in the heart (Zhu and He, 2015; Maejima et al., 2016; Sun et al., 2018). AMPK is considered to be cardioprotective and is associated with enhancing cardiomyocyte PQC, thus represents a potential therapeutic target if the exact mechanisms of action can be well-defined during cardiac disease.

#### Protein Kinase A (PKA)

Autophagic flux has been reported to be negatively and positively regulated by PKA phosphorylation of various targets (Torres-Quiroz et al., 2015). In a nutrient-rich environment Atg1 is phosphorylated by PKA which keeps Atg1 largely cytosolic and dissociated from the pre-autophagosomal structure (PAS), which inhibits autophagosome formation to suppress autophagy. During starvation Atg1 is dephosphorylated and localized to the PAS to facilitate autophagy (He and Klionsky, 2009). Atg13 is an essential subunit of the Atg1 autophagy initiation complex, which following phosphorylation by PKA at serine 437 will translocate away from the PAS. Conversely, inactivation of PKA induces autophagy by reducing the inhibitory phosphorylations of Atg13 (serines 344, 437, and 581) and Atg1 (serines 508 and 515) allowing Atg13 localization at the PAS (Budovskaya et al., 2005; Stephan et al., 2009). PKA phosphorylation of microtubule-associated protein 1 light chain 3 (LC3) at serine 12 inhibits its lipidation, a critical step involved in the incorporation of LC3 into autophagosomes, resulting in inhibition of autophagy (Cherra et al., 2010). In a model of diabetic cardiomyopathy, Lin28a overexpression improved cardiac function and prevented apoptosis by activating PKA/RhoA/ROCK2-dependent signaling and by upregulating autophagy (Sun et al., 2016). Taken together, these studies suggest that, much like the positive and negative reports of stimulating autophagy in cardiac disease, PKA can also elicit protective or detrimental responses. These disparities are likely due to compartmentalization of activation within specific micro-domains. Further studies are needed to tease out the differential PKA regulation of autophagy.

#### Protein Kinase G (PKG)

The cardioprotective abilities of PKG have been demonstrated in the setting of various cardiac diseases including PO, MI, ischemia/reperfusion injury and is the subject of many clinical trials as a heart failure treatment (Kokkonen-Simon et al., 2018; Dunkerly-Eyring and Kass, 2019; Pinilla-Vera et al., 2019; Oeing et al., 2020b). ER stress during cardiac aging and heart failure was abrogated by activating PKG by way of sildenafil treatment (PDE5 inhibition) in isoproterenol-induced or TAC-induced hypertrophy, or swimming exercise (Gong et al., 2013; Chang et al., 2020). A new signaling paradigm was revealed in early 2019 whereby PKG phosphorylates TSC2 (aka tuberin) at serine 1365, which results in mTORC1 inhibition but only in the presence of a pathological stimulus (Ranek et al., 2019). Intriguingly, oxidation of PKG impairs the ability of PKG to target TSC2 S1365 in response to a pathological stimulus adding a layer of complexity to this pathway (Oeing et al., 2020b). Phosphorylation of TSC2 serine 1365 or expression of a knock in phosho-mimetic (S1365E) mouse had no effect on basal mTORC1 activity or macroautophagy, however during cardiac PO mTORC1 hyperactivation was blocked resulting in stimulated autophagic flux, reduced cardiomyocyte hypertrophy, and increased cardiac function and lifespan (Ranek et al., 2019). These findings are unique as inhibition of mTORC1, including phosphorylation regulations are associated with a total inhibition of mTORC1 activity, whereas modulation of TSC2 serine 1365 does not influence mTORC1 activity at baseline but has a potent suppression (phosphorylated or phospho-mimetic) or exacerbation (unphosphorylated or phospho-silenced) in the presence of a pathological hypertrophy stimulus (Ranek et al., 2019; Oeing et al., 2020b). In a recent study, activating PKG with an GC-1 activator has also been shown to reduce oxidative stress and enhance autophagy in H9c2 cardiomyocytes (Zhao et al., 2020). In this study, H9c2 cardiomyocytes were pretreated with an GC-1 activator, followed by doxorubicin, a known inducer of oxidative stress. These cells had an increase in autophagosome formation, indicating an increase in autophagic flux due to the activation of PKG. Collectively these findings make PKG an attractive therapeutic target.

#### Phosphoinositide 3-Kinase (PI3K) and Protein Kinase B (PKB)

Activation of the class I phosphoinositide 3-kinase (PI3K)-protein kinase B (Akt) pathway negatively regulates autophagy during cardiomyocyte stress (Mellor et al., 2013). Macroautophagy has been associated with attenuation and exacerbation of cardiac disease following myocardial ischemia-reperfusion (I/R) injury, which appears to be due to the timing and the method of autophagy activation (Sciarretta et al., 2012b, 2018b). Excessive autophagy is deleterious to cardiac function, and as Li X. et al. (2018) demonstrated, stimulation of the PI3K/Akt/mTOR pathway reduced myocardial I/R injury by suppressing excessive autophagy. Similarly, in a 12-weeks of high fructose diet model Mellor et al. (2011) found that PI3K-Akt pathway suppression resulted in a dramatic upregulation of autophagy. A recent study noted that in mice subjected to aortic banding to induce left ventricular PO, there was enhanced Akt activity with reductions in autophagic flux and cardiac function, whereas inhibiting Akt activation increased autophagic flux an cardiac function (Xu et al., 2019). A study out of the Ghigo lab in 2018 investigated the role of PI3Kγ on autophagy in anthracycline induced cardiotoxicity (Li M. et al., 2018). Wild type mice treated with doxycycline exhibited increased PI3Kγ activity, Akt activation, and Ulk1 phosphorylation which were associated with reduced autophagic flux, cell survival, and cardiac function (Li M. et al., 2018). PI3Kγ-inhibited mice had suppressed Akt activation and Ulk1 phosphorylation which coupled to increased autophagic flux, cell survival, and cardiac function (Li M. et al., 2018). In addition to increasing mTORC1 activity, activation of the PI3K/Akt pathway also negatively regulates autophagy by phosphorylating and inhibiting the Ulk1/2 complex in diabetic hearts (Mellor et al., 2013). Additionally, Akt inhibits autophagy through phosphorylation of forkhead transcription factor (FoxO) protein to impede transcription of autophagy related genes *LC3*, gamma-aminobutyric acid receptor-associated protein-like 1 (*Gabarapl1*), and *Atg12* (Sengupta et al., 2009).

#### Extracellular Signal Regulated Protein Kinase (ERK)

Activation of the ERK regulates autophagy through maturation of the autophagic vacuoles (Corcelle et al., 2007). Treatment with trastuzumab (Herceptin), a monoclonal antibody commonly used as a therapy in HER2+ breast cancer, activated the ERK/mTORC1 pathway and inhibited autophagy in human primary cardiomyocytes resulting in increased production of ROS. Trastuzumab treatment interferes with cardiac HER2 signaling that leads to the phosphorylation of HER1-Y845/HER2-Y1248 and the activation of Erk. This in turn results in upregulation of the mTOR–Ulk1 pathway to mediate inhibition of autophagy in cardiomyocytes (Mohan et al., 2016). ERK is a negative regulator of TSC2, thereby activating mTORC1 which is known to suppress autophagy and accelerate cellular growth (Huang and Manning, 2008; Sciarretta et al., 2014, 2018a; Wang et al., 2019). Mitogen-activated protein kinase kinase (MEK) inhibition counteracted the protective effects of rapamycin on the induction of autophagy and attenuation of phenylephrine (PE)-induced cardiac hypertrophy, suggesting that ERK can also inhibit autophagy independent of mTORC1 (Gu et al., 2016). A highly selective inhibitor of MEK/ERK (U0126), reduced excessive autophagy, apoptosis, and infarct size in a model of cardiac I/R injury via the MEK/ERK/EGR-1 pathway (Wang et al., 2016). Studies in renal tubular epithelial cells and malignant human breast cells demonstrated that ERK positively regulates autophagy by facilitating the transition from LC3-I to LC3-II and increased production of beclin1 (Choi et al., 2010; Zeng et al., 2012). Further studies are needed to further determine the role of ERK regulation of autophagy in cardiac disease and identify the targets by which this occurs.

#### p38 MAPK

Similar to ERK, both activation and inhibition of autophagy have been reported with p38 MAPK stimulation (Webber and Tooze, 2010b). Autophagy-related (Atg) 5 is a crucial protein for autophagy to proceed. Atg5 is phosphorylated at threonine 75 by p38 MAPK leading to the inhibition of starvation-induced autophagy (Keil et al., 2013). The authors went on to create Atg5 phospho-silenced (threonine 75 to alanine, T75A) and phospho-mimetic (threonine 75 to glutamic acid, T75E) constructs, and these were used to transfect Atg5 deficient mouse embryonic fibroblasts (MEFs), thus the only Atg5 being expressed was the mutant constructs. Atg5 T75A MEFs contained autophagosomes in both the starved and serum fed conditions, suggesting enhanced basal autophagy, while the Atg5 T75E MEFs showed no autophagosome formation in either condition and had decreased autophagic flux (Keil et al., 2013). Another mechanism of inhibition is following a proinflammatory signal, whereupon p38α MAPK phosphorylates Atg1 (Ulk1) at serines 504 and 757 to disrupt its functional complex with Atg13 and reduce autophagy (He et al., 2018). Similarly, inhibition of p38 MAPK in myelogenous leukemic K562 cells increased beclin 1 expression and induction of autophagy (Colosetti et al., 2009). Alternatively, p38 MAPK was shown to phosphorylate the tumor suppressor protein p53 at serine 392, which enhanced its transcriptional activity causing increased expression of beclin1 resulting in stimulation of autophagy (Liu et al., 2009; Younce and Kolattukudy, 2010; Duan et al., 2011). Accordingly, in cultured neonatal rat cardiomyocytes exposed to 48 h of mechanical stretch and in mice following TAC, p38 MAPK inhibition caused a decrease in the autophagy marker LC3-II, suggesting a positive p38-autophagy relationship (Lin et al., 2014). Furthermore, Webber and Tooze provided evidence that the mAtg9–p38 MAPK interaction is important for autophagy and is regulated by the p38α activity (Webber and Tooze, 2010a). They identified p38 as a mAtg9 interacting protein and showed the interaction of mAtg9–p38 is required for starvation-induced mAtg9 trafficking and autophagosome formation, allowing the process of autophagy to proceed (Webber and Tooze, 2010a). The regulation of autophagy by p38 MAPK appears to be driven by the nature of the stimulus with potential sub-domain specificity.

#### c-Jun N-Terminal Kinase (JNK)

Increased autophagy has been reported with activation of c-Jun N-terminal Kinase (JNK), which has been attributed to a compensatory mechanism (Borsello et al., 2003; Jia et al., 2006; Li et al., 2006). JNK-dependent pathways are involved in the pathological mechanisms of myocardial hypertrophy and ischemia/reperfusion injury (He et al., 1999; Sun et al., 2012; Javadov et al., 2014). Other studies have shown that JNK activation is transient and varies depending on the severity and timing of oxidative stress during ischemia and reperfusion (Knight and Buxton, 1996; Laderoute and Webster, 1997; He et al., 1999; Fryer et al., 2001; Armstrong, 2004). JNK stimulates autophagy by phosphorylating anti-apoptotic protein B-cell lymphoma 2 (Bcl-2) (Chen et al., 2008; Wei et al., 2008a; Geeraert et al., 2010; Nopparat et al., 2010), which is typically bound to beclin1 thereby inhibiting autophagy (Pattingre et al., 2005), however once phosphorylated will dissociate from beclin1, resulting in the induction of autophagy (Wei et al., 2008b; Mrakovcic and Frohlich, 2018). JNK phosphorylation of Bcl-2 is associated with cell survival (Pattingre et al., 2009). JNK has also been shown to induce expression of pro-autophagic proteins SQSTM1 (p62), Atg5 and Atg7 in response to oxidative stress and oncogenic transformations as a method to enhance autophagy (Byun et al., 2009; Wong et al., 2010; Kim M. J. et al., 2011). JNK can also induce the expression of beclin1 via phosphorylation of c-Jun (Li et al., 2009; Ren et al., 2010). In contrast to the above mentioned pro-autophagic role of JNK, Xu et al. (2011) have demonstrated that targeted deletion of *JNK1*, *JNK2* and *JNK3* in neurons increases autophagy. Furthermore, in a diabetic cardiomyopathy model and in Angiotensin II-treated hearts, induction of miR-221 stimulated the transcription of JNK/c-Jun which resulted in depressed autophagy and exacerbated cardiac hypertrophy (Qian et al., 2017). Collectively, these studies demonstrate that much like the other MAP kinases, the regulatory role of JNK is context and stimuli dependent.

## Future Directions

Therapeutic strategies to enhance cardiac PQC are attractive, presenting a new direction for treatment of cardiac disease. Post-translational modifications, specifically phosphorylation and dephosphorylation, of key PQC proteins add selectivity and specificity to the protein degradation processes and provide a unique opportunity for pharmacological intervention. Indeed, some studies described here have demonstrated the cardioprotective potential of PQC enhancers. Here we have summarized the studies that investigated cardiac PQC (Supplementary Table 2). We also detailed other kinases that currently have not been linked to a protective pharmacological strategy, but may have immense potential. Phosphorylation of the proteins responsible for maintaining cardiac PQC during a pathological stimulus has added a new mechanism of regulation to this powerful system. With new drugs to target kinases and phosphatases, and advances in gene editing to create phospho-mimetic and -silenced proteins, the opportunities to manipulate these PQC systems are endless. Thus, elucidating the phosphorylations that regulate cardiac PQC is essential to advance our understanding and provide new therapeutic opportunities to interrogate cardiomyocyte PQC to treat cardiac disease.

## Author Contributions

SM and BD-E wrote the manuscript in consultation with GK. MR conceived, designed, and directed the writing. All authors provided critical feedback, interpretation, and contributed to the final manuscript.

## Conflict of Interest

BD-E and MR co-inventors on a patent application (PCT: 448070145WO1) that was filed in July 2018 (provisional filed in June 2017). The patent relates to the use of TSC2 (S1365/S1364) modifications for immunological applications. MR is a co-founder and shareholder of Meta-T Cellular, a start-up company that aims to develop applications of this intellectual property for immune therapy. The remaining authors declare that the research was conducted in the absence of any commercial or financial relationships that could be construed as a potential conflict of interest.

## Abbreviations

Akt: protein kinase B
Ambra1: activating molecule in beclin1-regulated autophagy 1
AMPK: AMP-activated protein kinase
AR: androgen receptor
ARG: Abl-related gene product
ASK1: apoptosis signal-regulating kinase 1
Atg: autophagy-related gene
CamKII: calcium/calmodulin dependent protein kinase
CASA: chaperone-assisted autophagy
CDK: cyclin dependent kinase
CHIP: carboxyl terminus of HSC70 interacting protein
c-IAP 1: cellular inhibitor of apoptosis protein 1
CK2: casein kinase 2
CMA: chaperone-mediated autophagy
CryAB: α B-crystallin
DRC: desmin-related cardiomyopathy
DYRK2: dual-specificity tyrosine-regulated kinase 2
ER: endoplasmic reticulum
ERAD: endoplasmic reticulum-associated degradation
ERK: extracellular signal regulated protein kinase
FGF: fibroblast growth factor
FGFR1: fibroblast growth factor receptor 1
FLNC: filamin-c
Foxo: forkhead transcription factor
HECT: homologous to E6-AP carboxyl terminus
HFpEF: heart failure with a preserved ejection fraction
HOP: HSP70-HSP90 organizing protein
HSP: heat shock protein
I/R: ischemia/reperfusion
IRS: insulin receptor substrate 1
JNK: c-JunN-terminal Kinase
MDM2: murine double minute 2
MI: myocardial infarction
MK2: mitogen kinase 2
MKK: mitogen-activated protein kinase kinase
mTORC1: mechanistic target of rapamycin complex 1
NEDD4: neural precursor cell expressed developmentally down-regulated protein 4
OA: okadaic acid
PAS: pre-autophagosomal structure
PCNA: proliferating cell nuclear antigen
PFI: proteasome functional insufficiency
PI3K: phosphoinositide 3-kinase
PINK1: PTEN-induced kinase 1
PKA: protein kinase A
PKC: protein kinase C
PKG: protein kinase G
PO: pressure overload
PP1 γ: protein phosphatase 1 γ
PP2A: protein phosphatase 2A
PQ: expanded polyglutamine
PQC: protein quality control
PTM: post-translational modification
RBR: ring between ring
RING: really interesting new gene
SCF, skp, cullin: F-box containing complex
SOD: super oxide dismutase
TAC: transverse aortic constriction
TFEB: transcription factor EB
TSC2: tuberous sclerosis complex 2
UBE2A: ubiquitin-conjugating enzyme E2A
UBE2J1: ubiquitin-conjugating enzyme E2J1
Ulk1: Unc-51 like autophagy activating protein kinase
UPS: ubiquitin proteasome system.
